# *Artemisia argyi*-Mediated Synthesis of Monodisperse Silver Nanoparticles as Components of Bioactive Nanofibrous Dressings with Dual Antibacterial and Regenerative Functions

**DOI:** 10.3390/jfb16070236

**Published:** 2025-06-27

**Authors:** Jiale Wang, Jiawei Guan, Xingyu Ma, Dongyang Zhao, Yongqiang Han, Dongdong Guo, Jialin Bai, Zisheng Guo, Xiaojun Zhang

**Affiliations:** 1 School of Medicine, Northwest University, Xi’an 710069, China; 2 Key Laboratory of Resource Biology and Biotechnology in Western China, Ministry of Education, Provincial Key Laboratory of Biotechnology, College of Life Sciences, Northwest University, Xi’an 710069, China

**Keywords:** silver nanoparticles, *Artemisia argyi*, biomaterial, antimicrobial, wound healing

## Abstract

The effective healing of chronic wounds requires balancing antimicrobial activity with tissue regeneration. In this study, we developed a novel, eco-friendly synthesis method using *Artemisia argyi* extract to produce silver nanoparticles (AgNPs), addressing toxicity concerns associated with conventional chemical synthesis methods. Through optimization of multiple synthesis parameters, monodisperse spherical AgNPs with an average diameter of 6.76 ± 0.27 nm were successfully obtained. Plant-derived compounds from *Artemisia argyi* extract acted as efficient mediators for both reduction and stabilization, yielding nanoparticles with high crystallinity. The synthesized AgNPs exhibited potent antibacterial activity against both Gram-negative and Gram-positive bacteria, with minimum inhibitory concentrations of 8 μg/mL against *Escherichia coli* and 32 μg/mL against *Staphylococcus aureus*, while maintaining high biocompatibility with L929 fibroblasts at concentrations ≤ 8 μg/mL. When integrated into polylactic acid/collagen type I (PLA/Col1) nanofibrous matrices, the optimized 0.03% AgNPs/PLA/Col1 dressing significantly accelerated wound healing in a diabetic rat model, achieving 94.62 ± 2.64% wound closure by day 14 compared to 65.81 ± 1.80% observed in untreated controls. Histological analyses revealed a dual-functional mechanism wherein controlled silver ion release provided sustained antibacterial protection, while concurrently promoting tissue regeneration characterized by enhanced collagen deposition, reduced inflammation, and increased neovascularization. This innovative approach effectively addresses critical challenges in diabetic wound care by providing simultaneous antimicrobial and regenerative functions within a single biomaterial platform.

## 1. Introduction

Diabetic wounds represent a significant global healthcare challenge, affecting an estimated 15–25% of more than 463 million people currently living with diabetes [1,2]. Patients with diabetes commonly experience significantly prolonged wound healing times and increased susceptibility to recurrent infections, primarily due to chronic hyperglycemia-induced microvascular complications and neuropathy [3,4]. This pathophysiological environment fosters persistent inflammation and elevates the risk of infection, predisposing patients to severe complications such as chronic ulcers and limb amputations [5]. Conventional clinical interventions frequently fail to adequately address the dual challenges of infection control and tissue regeneration, underscoring an urgent need for novel therapeutic strategies [6].

Recently, nanotechnology-based approaches have emerged as promising solutions to address complex involved in diabetic wound healing [7]. Among various nanomaterials, silver nanoparticles (AgNPs) have garnered considerable attention due to their nanoscale dimensions (1–100 nm), high surface area-to-volume ratio, and potent broad-spectrum antimicrobial properties [8]. The antibacterial, antiviral, and anti-inflammatory activities of AgNPs result from multiple mechanisms, including the disruption of bacterial cell membranes, inactivation of essential enzymes, and generation of reactive oxygen species (ROS) [9]. Recent studies have demonstrated the efficacy of AgNPs in accelerating wound closure and promoting angiogenesis, highlighting their efficiency for diabetic wound management [10,11].

AgNPs can be synthesized through various methods, each presenting distinct advantages and limitations [12]. Chemical and physical synthesis methods typically involve hazardous chemicals or sophisticated equipment, thus restricting their biomedical applicability [13,14]. Alternatively, biological synthesis methods provide an eco-friendly option by leveraging the natural reducing and stabilizing capabilities of biomolecules present in plant extracts, without generating harmful byproducts [15,16]. Such green synthesis approaches avoid the use of toxic reagents and utilize renewable resources rich in bioactive compounds—such as polyphenols, flavonoids, and terpenoids—which function effectively as reducing, stabilizing, and capping agents [17,18]. These naturally derived biomolecules facilitate the controlled formation of stable nanoparticles with tunable properties, often imparting additional therapeutic benefits derived from the intrinsic biological activities of the plant extracts utilized [19].

*Artemisia argyi* (*A. argyi*), a perennial herbaceous plant widely distributed throughout East Asia, presents an exceptional candidate for green AgNP synthesis due to its unique phytochemical composition [20]. *A. argyi* contains bioactive compounds, including flavonoids, volatile oils, polysaccharides, and triterpenoids, which collectively contribute to its antibacterial, anti-inflammatory, and antioxidant activities [21,22,23]. Combining the inherent medicinal properties of *A. argyi* with the antimicrobial efficacy of AgNPs offers a compelling strategy to develop advanced wound healing biomaterials. Preliminary investigations have already demonstrated the feasibility of synthesizing AgNPs using *A. argyi* leaf extract [24]. However, current research lacks comprehensive optimization of critical synthesis parameters necessary to precisely control nanoparticle characteristics. Additionally, systematic evaluations of cytotoxicity profiles and wound healing efficacy using diabetic models remain largely unexplored. Previous studies have indicated that smaller AgNPs typically exhibit enhanced antimicrobial activity due to their increased surface area-to-volume ratios and improved interactions with bacterial cell membranes [8,25]. Nevertheless, achieving optimal antimicrobial potency while ensuring colloidal stability and biocompatibility requires careful optimization of synthesis parameters [26].

In the field of wound healing, electrospun nanofiber dressings have emerged as a highly promising research focus due to their unique advantages. These dressings mimic the natural extracellular matrix through a biomimetic three-dimensional scaffold structure, leveraging their high specific surface area and interconnected porosity to create an ideal microenvironment for cell adhesion, migration, and proliferation, thereby accelerating neotissue formation [27]. Thanks to the precise control over fiber morphology afforded by electrospinning, bioactive agents such as antimicrobial peptides, epidermal growth factors, and collagen can be uniformly incorporated into the nanofibers to achieve sustained and controlled release [28]. This dual functionality not only effectively inhibits wound infection but also continuously promotes angiogenesis and collagen deposition [29]. Nanofiber dressings are particularly advantageous for chronic, hard-to-heal wounds, as they effectively prevent tissue dehydration and bacterial invasion, thereby establishing optimal conditions for wound repair.

The objective of this study was therefore to develop an optimized green synthesis protocol for producing *A. argyi*-mediated AgNPs tailored specifically for diabetic wound management. The influence of key reaction parameters on nanoparticle formation and characteristics was systematically investigated. Antimicrobial efficacy against Gram-negative (*Escherichia coli*) and Gram-positive (*Staphylococcus aureus*) bacteria was comprehensively evaluated, and the biocompatibility of synthesized AgNPs was assessed through in vitro fibroblast culture assays. To enhance potential clinical applicability, the optimized AgNPs were incorporated into electrospun polylactic acid/collagen type I (PLA/Col1) nanofibrous dressings, and their therapeutic efficacy was evaluated in a diabetic rat wound model. This work establishes a novel dual-functional biomaterial platform that effectively addresses the fundamental challenge in diabetic wound care by simultaneously providing antimicrobial protection and promoting tissue regeneration within a single integrated system.

## 2. Materials and Methods

### 2.1. Preparation of A. argyi Extract

Dried *A. argyi* leaves were obtained from Beijing Tongrentang (Group) Co., Ltd., China. The leaves were thoroughly washed with ultrapure water to remove dust and extraneous substances, then dried overnight in an oven at 40 °C. Subsequently, 2 g of finely cut dried leaves were added to 100 mL of ultrapure water and heated at 90 °C for 60 min. After cooling, the extract was filtered through Whatman No. 1 filter paper, sealed, and stored at 4 °C for subsequent use.

### 2.2. Identification of Extract Constituents

High-performance liquid chromatography coupled with mass spectrometry (HPLC-MS) is widely employed for the precise quantification and identification of chemical constituents in plant extracts, particularly phenolic compounds and flavonoids, owing to its high accuracy and sensitivity [30].

For constituent analysis, the prepared aqueous extract of *A. argyi* was filtered through Whatman No. 1 filter paper, and 1 mL of the filtrate was diluted tenfold. The diluted sample was further filtered through a 0.22 μm membrane to eliminate potential impurities and transferred to a suitable container for analysis. Chromatographic separation was performed using an ACQUITY ultra-performance liquid chromatography system equipped with a Waters ACQUITY HSS C18 column (2.1 × 100 mm, 1.8 μm). The eluate obtained from chromatographic separation was ionized and analyzed using an Agilent 5600 time-of-flight mass spectrometer equipped with an electrospray ionization source operating in negative ion mode to collect mass spectrometry data.

### 2.3. Synthesis and Optimization of AgNPs

In this study, *A. argyi* extract served as a natural reducing and stabilizing agent to synthesize AgNPs from silver nitrate (AgNO_3_, 99.9% purity, Macklin Biochemical Technology, Shanghai, China). Briefly, aqueous *A. argyi* extract was mixed with an aqueous solution of AgNO_3_, and the pH of the resulting mixture was adjusted using 0.1 mM hydrochloric acid (HCl, 37% *w*/*w*, Macklin Biochemical Technology, Shanghai, China) or 0.1 mM sodium hydroxide (NaOH, 98% purity, Macklin Biochemical Technology, Shanghai, China). Following pH adjustment, the mixture was continuously stirred and heated to induce nanoparticle formation. After synthesis, the nanoparticle-containing solution was centrifuged at 11,000 rpm for 30 min and washed twice with anhydrous ethanol. Finally, the purified nanoparticles were dried in a vacuum oven at 60 °C for 12 h to obtain dry AgNP powder.

To optimize the green synthesis process, several critical parameters were systematically investigated, including volume ratios of *A. argyi* extract to AgNO_3_ solution (1:1, 1:2, 1:3, 1:4, and 1:5), AgNO_3_ concentrations (2, 4, 6, 8, and 10 mM), *A. argyi* extract concentrations (1, 2, 4, 6, and 8 mg/mL), solution pH values (5, 6, 7, 8, and 9), reaction durations (15, 30, 45, 60, and 75 min), and heating temperatures (25, 40, 60, 80, and 90 °C). Through the rigorous optimization of these parameters, stable AgNPs with controlled size and morphology were successfully synthesized.

### 2.4. Characterization of AgNPs

The size, morphology, and surface topography of the synthesized AgNPs were analyzed using transmission electron microscopy (TEM; JEOL JEM-200, Kyoto, Japan). The crystalline structure was determined by X-ray diffraction (XRD; SmartLab SE, Kyoto, Japan), and elemental composition was characterized using energy-dispersive X-ray spectroscopy (EDS; JEOL JEM-200, Kyoto, Japan). Functional groups present on the synthesized AgNPs were identified by Fourier-transform infrared spectroscopy (FTIR; TENSOR 27, Bruker, Germany), with scans performed in the spectral range of 4000–400 cm^−1^ [31]. Additionally, the hydrodynamic size distribution and polydispersity index (PDI) of AgNPs in aqueous suspension were determined using dynamic light scattering (DLS) analysis on a Malvern Panalytical-Zetasizer Nano S90 (Malvern Instruments Ltd., Worcestershire, UK) at 25 °C. Zeta potential measurements were also conducted to assess the surface charge and colloidal stability of the AgNPs.

### 2.5. Cell Culture and Cytotoxicity Evaluation

Rat fibroblast cells (L929 cell line, Procell, Wuhan, China) were cultured in Dulbecco’s Modified Eagle’s Medium (DMEM; HyClone, Logan, UT, USA), supplemented with 10% fetal bovine serum (FBS; Sijiqing Bioengineering, Hangzhou, China) and 1% penicillin–streptomycin solution (Beyotime Biotechnology, Shanghai, China). Cells were maintained at 37 °C in a humidified incubator containing 5% CO_2_.

Cytotoxic effects of AgNPs on L929 cells were assessed using the Cell Counting Kit-8 (CCK-8) assay (Beyotime Biotechnology, Shanghai, China). Briefly, cells were seeded into 96-well plates at a density of 1 × 10^4^ cells/well and treated with varying concentrations of AgNPs (1–256 μg/mL) for 24 h. Subsequently, 10 μL of CCK-8 reagent was added to each well, and the plates were incubated for an additional 2 h. The absorbance at 450 nm was then measured using a multifunctional microplate reader (BioTek Synergy H1, Agilent, Santa Clara, CA, USA).

### 2.6. Antibacterial Activity of AgNPs

#### 2.6.1. Preparation of Bacterial Suspensions

The antibacterial activity of synthesized AgNPs was evaluated against Gram-positive (*S. aureus*, ATCC 25923) and Gram-negative (*E. coli*, ATCC 25922) bacteria. A single colony from Luria–Bertani (LB) agar medium was inoculated into LB broth and incubated for 12 h. Subsequently, 1% (*v*/*v*) of the bacterial culture was transferred into fresh LB broth and activated by incubation for an additional 3 h. The bacterial suspension was then adjusted to an optical density at 600 nm (OD_600_) of 0.5 for subsequent antibacterial assays.

#### 2.6.2. Growth Curve Analysis

Bacterial suspensions (90 μL, approximately 1 × 10^6^ CFU/mL) were added into each well of sterile 96-well plates. Experimental groups were treated with 10 μL AgNP solutions at concentrations ranging from 1 to 64 μg/mL. Blank control wells received 10 μL sterile LB medium, while positive control wells received 10 μL antibiotic solutions (2 mg/mL gentamicin for *E. coli* or ampicillin for *S. aureus*). The OD_600_ values were recorded at intervals of 2, 4, 6, 8, 10, 12, and 24 h using a microplate reader.

#### 2.6.3. Minimum Inhibitory Concentration (MIC) and Minimum Bactericidal Concentration (MBC) Determination

The MIC and MBC of the AgNPs were determined against both bacterial strains. Bacterial suspensions (1 × 10^6^ CFU/mL) were treated with AgNP solutions at concentrations ranging from 1 to 64 μg/mL. Negative controls (LB broth only) and positive controls (gentamicin for *E. coli* and ampicillin for *S. aureus*) were included. After 24 h incubation, MIC was defined as the lowest concentration of AgNPs resulting in no visible bacterial growth, as confirmed by OD_600_ measurements. For MBC determination, bacterial suspensions from wells showing no visible growth were diluted to approximately 1 × 10^4^ CFU/mL, plated onto LB agar, and incubated at 37 °C for 24 h. MBC was recorded as the lowest concentration of AgNPs yielding fewer than five colonies.

#### 2.6.4. Inhibition Zone Assay

Bacterial suspensions (approximately 1 × 10^4^ CFU/mL) were uniformly spread onto LB agar plates. Sterile filter paper disks were saturated with different concentrations of AgNP solutions (16, 32, 64, and 128 μg/mL), antibiotics (gentamicin for *E. coli* and ampicillin for *S. aureus*), 1 mM AgNO_3_ solution, or *A. argyi* leaf extract. Plates were incubated at 37 °C for 24 h, after which the diameters of inhibition zones were measured to evaluate antibacterial efficacy.

### 2.7. Preparation of AgNPs/PLA/Col1 Electrospun Dressings

PLA and Col1 were dissolved in hexafluoroisopropyl alcohol at a mass ratio of 1:1. Optimized AgNPs were incorporated into the PLA/Col1 solution at concentrations of 0%, 0.03%, and 0.05% (*w*/*v*). Electrospinning was performed using a voltage of 13 kV, a solution flow rate of 1 mL/h, and a needle-to-collector distance of 12–13 cm. Following electrospinning, the resulting nanofibrous mats were vacuum-dried at 37 °C for 48 h and subsequently cut into disks (10 mm in diameter) for further experiments. The morphology of electrospun nanofibers was characterized using scanning electron microscopy (SEM).

### 2.8. Cytotoxicity and Antibacterial Performance of Electrospun Fibers

#### 2.8.1. Cytotoxicity Evaluation of Electrospun Fibers

Electrospun fiber disks were placed into 24-well plates and sterilized via ozone and ultraviolet (UV) irradiation for 30 min. Subsequently, L929 fibroblast cells were seeded onto the electrospun scaffolds at a density of 1 × 10^5^ cells per well and cultured at 37 °C in a humidified incubator containing 5% CO_2_ for 1, 4, and 7 days. At each predetermined time point, the culture medium was replaced with 200 μL DMEM containing 5% CCK-8 solution. After incubation for an additional 2 h, supernatant absorbance was measured at 450 nm using a microplate reader to evaluate cell viability.

#### 2.8.2. Antibacterial Performance Evaluation

Sterilized AgNPs/PLA/Col1 electrospun disks were initially incubated in 2 mL LB broth for 24 h with continuous shaking. After removing the disks, bacterial suspension (20 μL, approximately 1 × 10^6^ CFU/mL) was added to the conditioned medium and incubated at 37 °C. Aliquots were collected at 0, 4, 8, and 12 h, and their OD_600_ was measured using a microplate reader to assess bacterial growth and evaluate the antibacterial efficacy of the electrospun fibers.

### 2.9. Evaluation of Wound Healing Ability In Vivo

#### 2.9.1. Establishment of Diabetic Rat Model

All animal experiments were conducted in accordance with the guidelines established by Northwest University of China and the National Institutes of Health Guidelines for the Care and Use of Laboratory Animals (No: NWU-AWC-20230421R). Four-week-old male Sprague–Dawley rats (approximately 85 g) were obtained from the Laboratory Animal Center of Xi’an Jiaotong University and housed under standard conditions (12 h light/dark cycle, temperature 20–25 °C, humidity 40–70%).

Animals were maintained on a purified diet for 4 weeks prior to diabetes induction, which was achieved via intraperitoneal injection of streptozotocin (STZ, 35 mg/kg). Random blood glucose levels were monitored at days 3, 7, and 14 post-STZ administration. The successful establishment of the diabetic model was confirmed when blood glucose levels consistently exceeded 16.67 mmol/L for three consecutive days. Throughout the experimental period, physiological parameters—including activity, fur condition, food and water intake, and body weight—were continuously monitored.

#### 2.9.2. Preparation of Diabetic Rat Wound Model

Following a fasting period of 16 h, rats were anesthetized by intraperitoneal injection of 1% pentobarbital sodium solution (35 mg/kg). The dorsal area was shaved, and the skin was sterilized with medical iodine solution. Two full-thickness wounds (10 mm diameter) were surgically created on the dorsal region of each rat. Sixteen diabetic rats were used to create a total of 32 wound sites for analysis. To minimize inter-individual variation, each rat received two different treatments on separate wounds. Eight rats had one wound treated as control (untreated) and the other wound treated with PLA/Col1 dressing. The remaining 8 rats had one wound treated with 0.03% AgNPs/PLA/Col1 and the other with 0.05% AgNPs/PLA/Col1. Scheduled endpoints were established at day 7 (8 rats sacrificed, providing 4 samples per treatment group) and day 14 (remaining 8 rats sacrificed, providing 4 samples per treatment group). Post-surgery, animals were individually housed without the administration of antibiotics or analgesics.

Wound healing progression was monitored by capturing digital images on days 0, 3, 7, and 14. Wound areas were measured using ImageJ 1.54f software, and the wound closure percentage was calculated according to the following formula:

(1) $$Wound\;contraction\;percentage=\frac{S_{initial}-S_{current}}{S_{initial}}\times 100\%$$

where S_initial_ represents the initial wound area, which is the original wound area obtained through digital photography on the 0th day of the experiment, in square millimeters (cm^2^). S_current_ represents the wound area at the current time point, which is the real-time residual wound area measured by Image J software on the 3rd, 7th, and 14th day after injury, with the same unit as the initial area.

On day 14 post-surgery, tissue specimens were collected for histological analysis. Hematoxylin and eosin (H&E) staining was employed to assess general wound healing and granulation tissue formation, Masson’s trichrome staining was used to evaluate collagen deposition, and CD31 immunohistochemical staining was conducted to detect neovascularization.

### 2.10. Statistical Analyses

All experiments were performed with at least three independent replicates, and data are presented as mean ± standard deviation (SD). Statistical significance was assessed using one-way analysis of variance (ANOVA) followed by Tukey’s post hoc test for multiple comparisons, with *p* < 0.05 considered statistically significant. Data visualization and statistical analyses were conducted using Origin 2022, Image J 1.54f, and NanoMeasurer 1.2 software.

## 3. Results and Discussion

### 3.1. Characterization of A. argyi Extract by HPLC-MS Analysis

**Figure 1:**
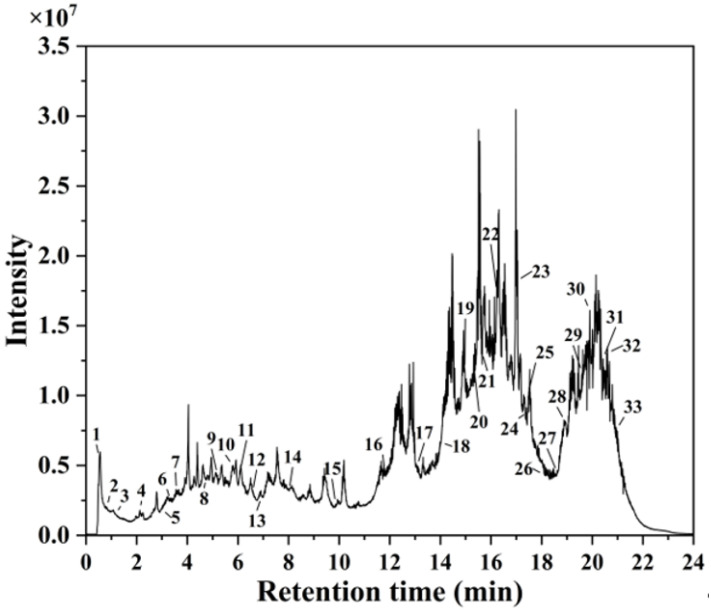
HPLC-MS chromatogram of *A. argyi* extract.

HPLC-MS analysis identified 33 distinct secondary metabolites in the *A. argyi* extract (Figure 1). These compounds primarily consisted of flavonoids, terpenoids, carboxylic acids, glycosides, and phenolic acids. Key flavonoids included kaempferol 3-apiosyl-(1→2)-galactoside, quercetin 3-*O*-glucuronide, isorhamnetin-3-*O*-rutinoside, diosmetin, eupatilin, baicalin, chrysin, and acacetin. Similar flavonoid derivatives have previously been identified in *Jasminum officinale* L. leaf extracts utilized for AgNP synthesis [32]. The extract also contained carbohydrate derivatives and glycosides, which served effectively as capping agents, stabilizing the synthesized AgNPs and preventing particle agglomeration [33]. Additionally, fatty acids—including 9-HODE, sanggenol O, and 12,13-DiHOME—acted as primary reducing agents for Ag^+^ ions, consistent with previous studies demonstrating the efficacy of fatty-acid-capped AgNPs in antimicrobial applications [34].

### 3.2. Optimization of AgNP Synthesis Conditions

**Figure 2:**
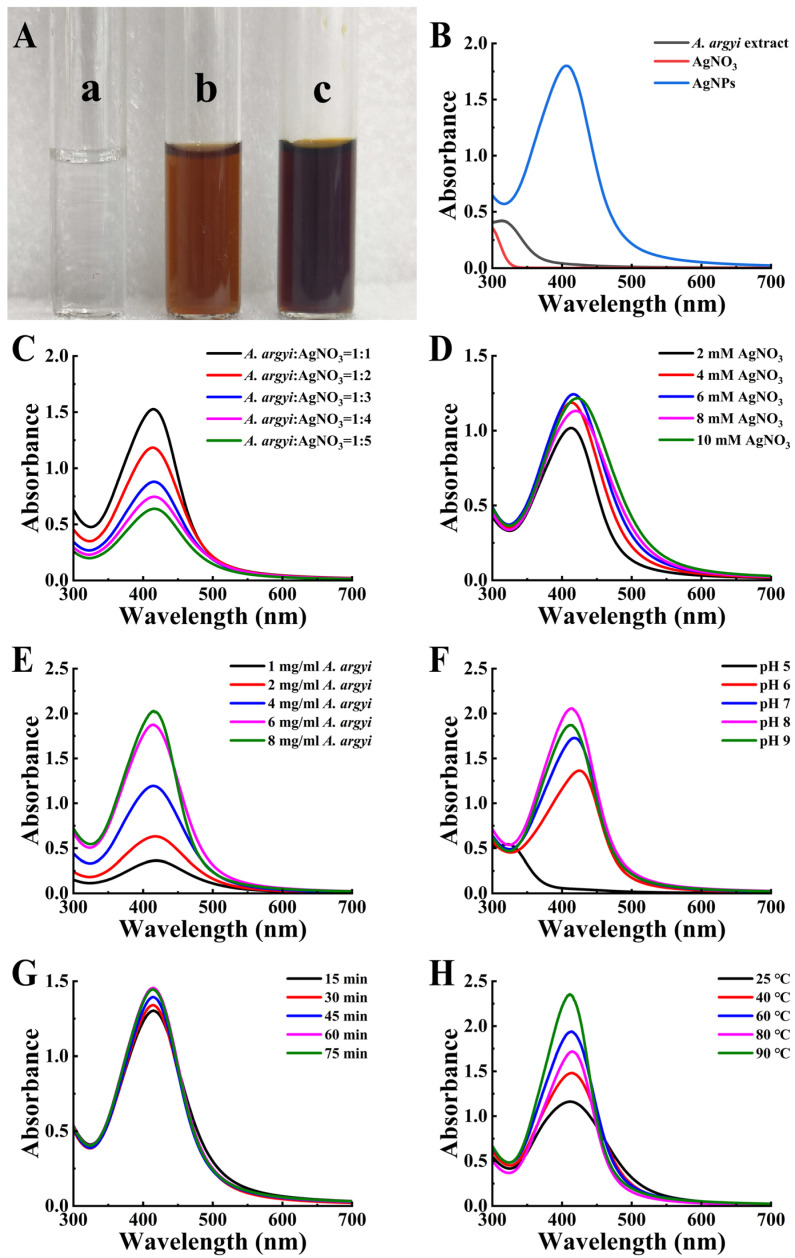
Synthesis and optimization of AgNPs. (**A**) Color transition of the reaction mixture indicating AgNP synthesis. (**a**) Initial AgNO_3_ solution; (**b**) immediate reaction mixture; (**c**) final AgNP solution. (**B**) UV-vis spectra of AgNPs. (**C**–**H**) Optimization parameters: (**C**) *A. argyi* extract-to-AgNO_3_ volume ratio, (**D**) AgNO_3_ concentration, (**E**) *A. argyi* concentration, (**F**) pH, (**G**) reaction time, and (**H**) reaction temperature.

Upon addition of *A. argyi* extract to AgNO_3_ solution, a visible color change from clear to reddish-brown occurred (Figure 2A), indicating successful AgNP formation via surface plasmon resonance (SPR) effects (Figure 2B) [35]. To achieve optimal synthesis conditions for producing AgNPs with targeted physicochemical properties, multiple reaction parameters were systematically investigated and optimized (Table 1).

#### 3.2.1. Effect of Volume Ratio, Concentration, and pH

The volume ratio of *A. argyi* extract to AgNO_3_ solution significantly influenced nanoparticle characteristics. A volume ratio of 1:1 yielded the highest AgNP production and resulted in smaller, more uniform particles (Figure 2C). Optimization of the AgNO_3_ concentration indicated that 6 mM provided optimal results. Increasing the AgNO_3_ concentration from 2 mM to 6 mM enhanced the availability of Ag^+^ ions, as evidenced by increased SPR absorbance (Figure 2D). However, further increases to 8–10 mM resulted in decreased absorbance intensity and peak broadening, indicative of particle aggregation or increased particle size [36]. Regarding *A. argyi* extract concentration, the SPR absorbance intensity increased proportionally alongside increasing extract concentration, accompanied by a narrowing of the absorbance peak, suggesting reduced nanoparticle size (Figure 2E). The optimal extract concentration was determined to be 8 mg/mL, as higher concentrations introduced impurities and destabilized the synthesized nanoparticles [37].

Reaction pH profoundly influenced nanoparticle morphology by modulating the surface charge and reducing the potential of biomolecules involved in synthesis [38]. Under alkaline conditions (pH > 7), the SPR absorption intensity increased, yielding smaller and more uniform nanoparticles (Figure 2F) [39]. Specifically, pH 8 provided the optimal environment for maximizing nanoparticle yield and uniformity by modulating the surface charge and reducing potential of biomolecules involved in synthesis. At pH 8, the deprotonation of hydroxyl groups in flavonoids and phenolic compounds enhances their reducing capacity for Ag^+^ ions, while maintaining colloidal stability of the synthesized nanoparticles.

#### 3.2.2. Effect of Reaction Time and Temperature

Analysis of reaction time demonstrated that nanoparticle yield and SPR peak absorbance initially increased with prolonged durations (Figure 2G). The optimal reaction duration was identified as 60 min, as longer reaction times induced particle aggregation, compromising particle uniformity and stability. During the preliminary screening phase, temperature was maintained at 50 °C based on literature precedents for plant extract-mediated synthesis. Investigation into the effects of reaction temperature revealed that a temperature of 60 °C produced the most symmetric SPR peak, indicative of uniform particle dispersion and optimal particle size distribution (Figure 2H). Although elevated temperature promotes activation of biomolecules as reducing agents, temperatures exceeding 60 °C risked biomolecule degradation and compromised nanoparticle stability [40].

Based on these systematic investigations, the optimized synthesis conditions were established as follows: *A. argyi* extract-to-AgNO_3_ ratio of 1:1, AgNO_3_ concentration of 6 mM, *A. argyi* extract concentration of 8 mg/mL, reaction pH of 8, reaction time of 60 min, and reaction temperature of 60 °C (not 50 °C as used in preliminary screening). This systematic one-factor-at-a-time optimization approach successfully identified optimal synthesis conditions that yielded monodisperse, spherical AgNPs with narrow size distribution. However, we acknowledge that this optimization strategy may not fully capture potential synergistic interactions between parameters. While our approach effectively maximized individual parameter contributions to nanoparticle formation, future investigations could employ response surface methodology or factorial experimental designs. These approaches would comprehensively evaluate parameter interdependencies and identify potential synergistic effects that might further enhance nanoparticle characteristics and synthesis efficiency.

### 3.3. Comprehensive Characterization of Synthesized AgNPs

#### 3.3.1. Morphological and Elemental Analysis

**Figure 3:**
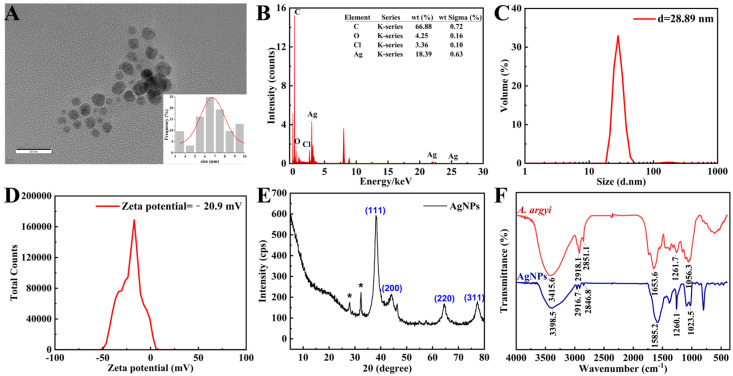
Structural and chemical characterization of AgNPs. (**A**) TEM image of AgNPs (inset: size distribution histogram). (**B**) EDS spectrum confirming the presence of elemental Ag (3 keV peak). (**C**) Size distribution of AgNPs measured by DLS. (**D**) Zeta potential analysis of AgNPs via DLS. (**E**) XRD pattern indexed to face-centered cubic (FCC) silver. (**F**) FTIR spectra of the *A. argyi* extract and AgNPs. * in (**E**) denotes “AgCl impurities originating from plant-based synthesis materials”.

TEM imaging revealed densely distributed, predominantly spherical AgNPs within a narrow size range of 3–10 nm, exhibiting an average diameter of 6.76 ± 0.27 nm (Figure 3A). The nanoscale dimensions observed significantly enhance antimicrobial efficacy by maximizing the available surface area for bacterial interaction [41]. EDS analysis confirmed elemental silver as the primary composition, exhibiting the characteristic silver (Ag) signal at approximately 3 keV (Figure 3B) [42]. The detection of trace carbon and oxygen signals suggests the presence of organic compounds derived from *A. argyi* extract, acting as effective capping and stabilizing agents [43].

#### 3.3.2. Characterization of Dynamic Dimensions and Colloidal Potential

The DLS analysis revealed that the average particle size of the AgNPs was 28.89 nm (Figure 3C), with a polydispersity index of 0.439 (Figure 3D), suggesting a relatively narrow size distribution with moderate polydispersity [44]. The unimodal size distribution indicates the absence of significant secondary populations (e.g., aggregates or impurities), while the PDI value reflects a certain degree of size heterogeneity. Such a distribution provides a stable foundation for the application of silver nanoparticles in biomedical and catalytic fields. Additionally, the zeta potential measurement yielded a value of −20.9 mV, indicative of strong electrostatic repulsion between particles, thereby confirming the long-term colloidal stability of the nanoparticles. The negative surface charge is likely attributed to organic molecules derived from the plant extract adsorbed on the nanoparticle surface [45].

When comparing DLS results with TEM-measured particle sizes, discrepancies arise from fundamental differences in measurement principles. TEM employs electron beams to directly image the physical morphology of dried nanoparticles on ultrathin samples, providing size measurements unaffected by the surrounding hydration layer. In contrast, DLS measures the hydrodynamic diameter of nanoparticles suspended in liquid, which includes the nanoparticle core plus the adsorbed hydration shell, resulting in larger size values [46].

#### 3.3.3. Crystallographic and Molecular Characterization

XRD analysis exhibited characteristic diffraction peaks at 38.33° (111), 46.43° (200), 64.66° (220), and 77.55° (311), confirming the face-centered cubic crystalline (fcc) structure of the synthesized AgNPs (Figure 3E), consistent with previous reports on plant-mediated AgNP synthesis [47]. Additional diffraction peaks corresponded to AgCl formation, likely due to chloride ions inherently present in the *A. argyi* extract. These AgCl species may further enhance antibacterial properties through synergistic antimicrobial mechanisms [48].

FTIR spectrometer was employed to identify the key functional groups involved in the reduction in Ag^+^ ions and stabilization of AgNPs (Figure 3F). The observed redshift of the O–H stretching vibration from 3415.6 cm^−1^ in the pure *A. argyi* extract to 3398.5 cm^−1^ in the synthesized AgNP spectrum confirmed the active role of hydroxyl groups in Ag^+^ ion reduction through electron donation [49]. Characteristic bands at approximately 2916.7 cm^−1^ and 2846.8 cm^−1^ (C–H stretching vibrations) and 1582.2 cm^−1^ (C=O stretching vibration) indicated the presence of alkane and carbonyl groups, respectively, derived from plant constituents and functioning as effective capping agents [50]. Collectively, the FTIR spectral data confirmed that flavonoids and phenolic compounds present in the extract simultaneously mediated both nanoparticle reduction and stabilization. Specifically, hydroxyl groups facilitated the reduction in Ag^+^ ions to metallic Ag, while carbonyl and amine functionalities prevented nanoparticle aggregation through effective surface coordination [43].

### 3.4. Biological Activity Assessment

#### 3.4.1. Cytotoxicity Evaluation of AgNPs

**Figure 4:**
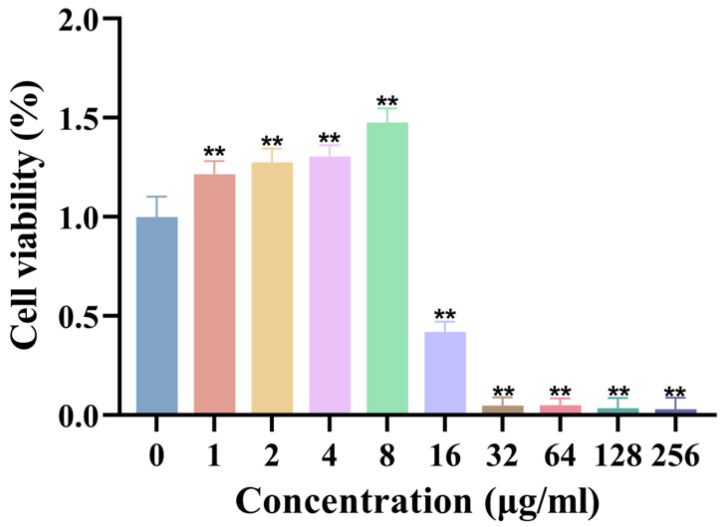
Cytotoxicity of AgNPs on L929 fibroblasts. Cell viability (%) after 24 h exposure to AgNPs (*n* = 8). ** *p* < 0.01.

The biocompatibility of synthesized AgNPs was assessed using the L929 fibroblast cell line. The results demonstrated that AgNPs exhibited no significant cytotoxicity to fibroblasts at concentrations ≤ 8 μg/mL, maintaining cell viability comparable to that of control groups (Figure 4). However, cell viability was significantly reduced at concentrations ≥ 16 μg/mL, establishing a clear concentration-dependent cytotoxicity profile. These findings underscore the importance of precise dosage control to achieve a balance between robust antimicrobial efficacy and cellular safety in biomedical applications.

#### 3.4.2. Comprehensive Antibacterial Activity Analysis

**Figure 5:**
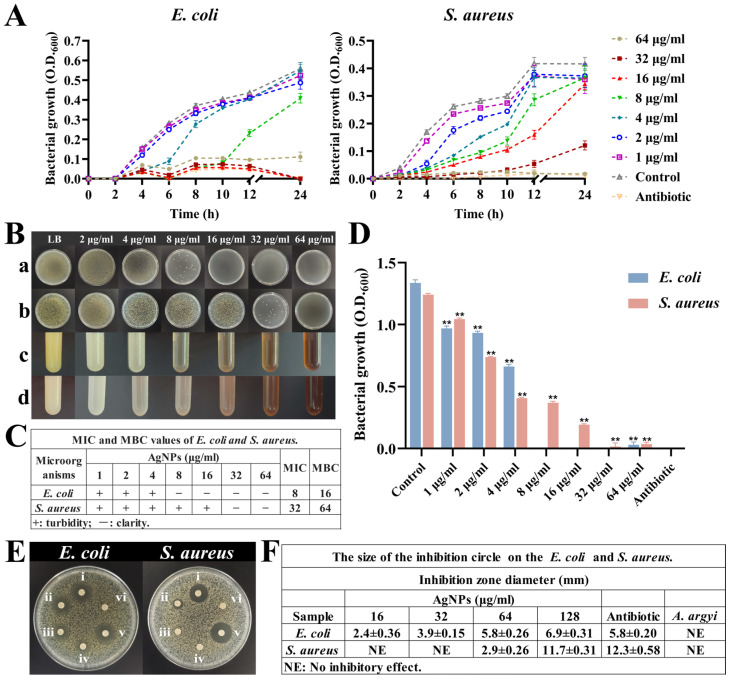
Antibacterial activity of AgNPs. (**A**) Growth curves of *E. coli* and *S. aureus* under different concentrations of AgNPs. (**B**) Bacterial growth under different concentrations of AgNPs. (**a**,**b**) Plates grown with *E. coli* and *S. aureus* at different concentrations of AgNPs. (**c**,**d**) Turbidity of *E. coli* and *S. aureus* growth at different concentrations of AgNPs. (**C**) MIC and MBC values of *E. coli* and *S. aureus*. (**D**) Growth of both bacteria at 24 h under different concentrations of AgNPs, ** *p* < 0.01. (**E**) Inhibition zones: (i–iv) AgNPs at 128, 64, 32, and 16 μg/mL; (v) antibiotic control; (vi) *A. argyi* extract alone. (**F**) The size of inhibition circle.

The antibacterial evaluation revealed potent antimicrobial activity of synthesized AgNPs against both Gram-negative (*E. coli*) and Gram-positive (*S. aureus*) bacteria. Growth curve analyses demonstrated significant bacteriostatic effects at AgNP concentrations ranging from 4 to 64 μg/mL during the initial 6 h incubation period (Figure 5A). Notably, the growth of *E. coli* was effectively suppressed at concentrations of 16–64 μg/mL, whereas *S. aureus* required higher concentrations (32–64 μg/mL) for comparable suppression.

The MICs for synthesized AgNPs were determined to be 8 μg/mL for *E. coli* and 32 μg/mL for *S. aureus* (Figure 5B–D). These MIC values closely align with previously reported values, where similarly sized AgNPs exhibited MICs of approximately 6 μg/mL against *E. coli* and 25–50 μg/mL against various other bacterial strains [51]. Additionally, MBC analyses determined bactericidal thresholds of 16 μg/mL for *E. coli* and 64 μg/mL for *S. aureus* (Figure 5B(a,b)), confirming the transition from bacteriostatic to bactericidal effects at higher nanoparticle concentrations [52,53].

Inhibition zone assays further validated these findings, demonstrating a direct correlation between AgNP concentration and zone diameter (Figure 5E,F). Specifically, *E. coli* exhibited larger inhibition zones (5.8 ± 0.26 mm at 64 μg/mL) compared to *S. aureus* (2.9 ± 0.26 mm at 64 μg/mL), a result reflective of structural differences in bacterial cell wall composition and susceptibility [54].

The potent antibacterial effects of AgNPs involve multiple mechanisms: Ag^+^ ions released from nanoparticles disrupt bacterial cell wall integrity. AgNPs themselves alter membrane permeability, and both Ag^+^ ions and AgNPs damage bacterial DNA replication machinery either directly or through the induction of ROS [55,56]. This multi-targeted antibacterial mechanism significantly reduces the potential for bacterial resistance development, highlighting the clinical relevance and long-term efficacy of these synthesized nanoparticles.

### 3.5. Development and Characterization of AgNPs/PLA/Col1 Nanofiber Dressings

**Figure 6:**
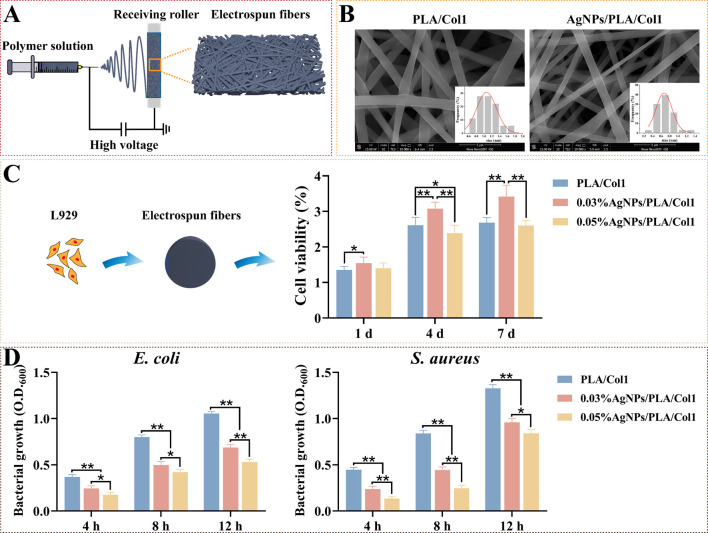
Electrospun AgNPs/PLA/Col1 dressings. (**A**) Schematic representation of electrospinning using AgNPs, PLA, and Col1. (**B**) SEM images of nanofibers (scale bar: 5 μm). (**C**) L929 viability after 4 days (*n* = 8). (**D**) Antibacterial efficacy against *E. coli* and *S. aureus* (*n* = 5), * *p* < 0.05, ** *p* < 0.01.

To translate our optimized AgNPs into clinically relevant wound healing platforms, we fabricated composite nanofiber dressings via electrospinning (Figure 6A). This technique generates biomimetic matrices closely resembling the natural extracellular matrix, thereby facilitating cellular infiltration and promoting tissue regeneration [57]. SEM analysis revealed smooth, uniformly oriented fibers arranged into an interconnected three-dimensional porous network (Figure 6B). Notably, the incorporation of AgNPs significantly reduced fiber diameter from 1.06 ± 0.03 μm (PLA/Col1) to 0.67 ± 0.02 μm (AgNPs/PLA/Col1), an effect attributed to increased electrospinning solution conductivity imparted by AgNP inclusion [58].

CCK-8 cytocompatibility assays demonstrated that 0.03% AgNPs/PLA/Col1 dressings maintained high fibroblast viability comparable to the pure PLA/Col1 control throughout a 4-day incubation period. In contrast, the 0.05% AgNPs/PLA/Col1 formulation exhibited substantial cytotoxicity by day 4, highlighting dose-dependent biocompatibility (Figure 6C). Antibacterial evaluations indicated that both AgNP-loaded dressings significantly inhibited bacterial growth of *E. coli* and *S. aureus* compared to control dressings lacking AgNPs, with the 0.05% formulation demonstrating superior antibacterial activity (Figure 6D).

### 3.6. In Vivo Wound Healing Efficacy in the Diabetic Rat Model

#### 3.6.1. Wound Closure Kinetics

**Figure 7:**
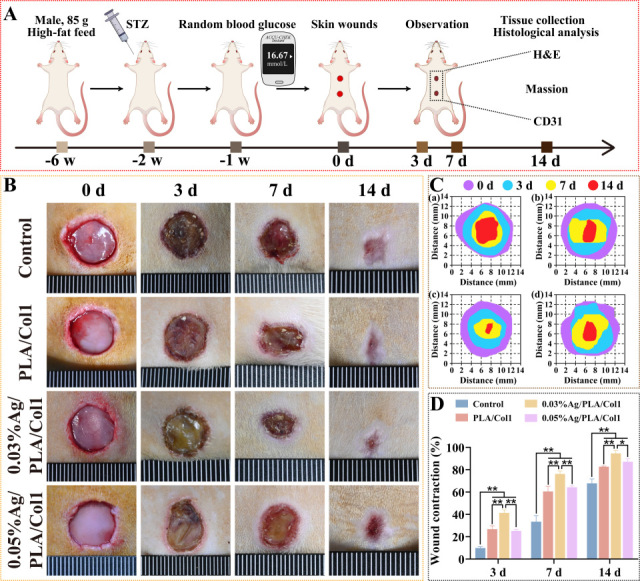
In vivo wound healing in diabetic rats. (**A**) Schematic of the diabetic wound model. (**B**) Wound images over 14 days. (**C**) Wound tracings: (**a**) control, (**b**) PLA/Col1, (**c**) 0.03% AgNPs/PLA/Col1, and (**d**) 0.05% AgNPs/PLA/Col1. (**D**) Wound closure rates (*n* = 4). * *p* < 0.05, ** *p* < 0.01 vs. control.

To evaluate therapeutic efficacy, a full-thickness excisional wound model was established in diabetic rats (Figure 7A). By day 3 post-operation, scab formation was observed consistently across all experimental groups. Notably, the 0.03% AgNPs/PLA/Col1 dressing group demonstrated significantly accelerated wound closure compared to all other treatment groups, achieving 94.62 ± 2.64% wound contraction by day 14 (Figure 7B–D). In comparison, the untreated control, PLA/Col1, and 0.05% AgNPs/PLA/Col1 groups exhibited wound closure rates of 65.81 ± 1.80%, 82.72 ± 0.56%, and 87.17 ± 2.66%, respectively.

The superior wound healing performance observed in the 0.03% AgNPs/PLA/Col1 group is attributable to the dressing’s synergistic dual functionality. The biomimetic PLA/Col1 nanofiber scaffold effectively promotes cellular migration, extracellular matrix deposition, and tissue remodeling, while the precisely controlled release of AgNPs inhibits microbial colonization and modulates inflammation. Collectively, these features create an optimal microenvironment conducive to efficient diabetic wound healing and tissue regeneration.

#### 3.6.2. Histological and Immunohistochemical Analysis

**Figure 8:**
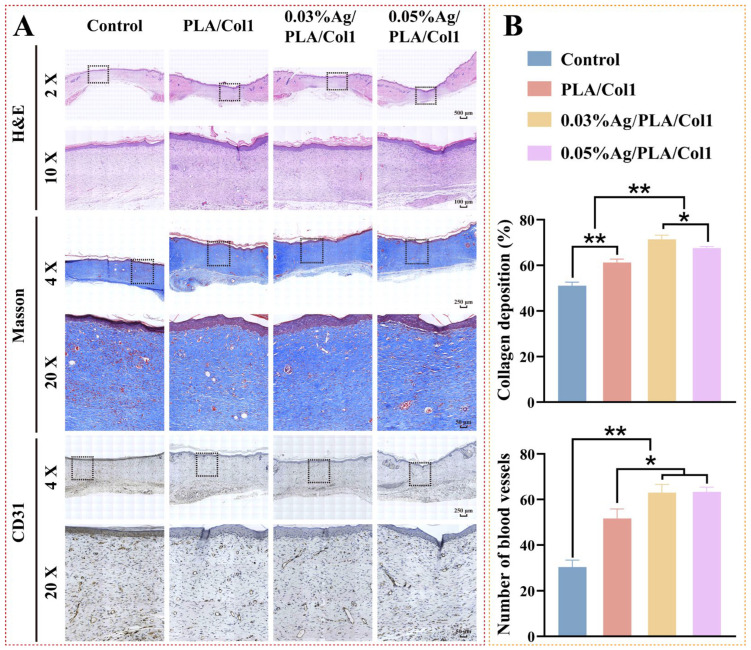
Histological analysis of healed wounds. (**A**) Representative images of H&E staining, Masson’s trichrome staining (collagen, blue), and CD31 immunohistochemical staining on day 14. (**B**) Quantification of collagen deposition (%) and CD31+ vessel density (*n* = 4). * *p* < 0.05, ** *p* < 0.01 vs. control.

Histological evaluation via H&E staining performed on day 14 post-operation provided insights into wound healing profiles across experimental groups (Figure 8A). Notably, the 0.03% AgNPs/PLA/Col1 group exhibited minimal inflammatory cell infiltration, indicative of effective infection control and reduced inflammation within diabetic wounds [59]. Although all treated groups demonstrated improved epithelial regeneration relative to the untreated control—which exhibited only a thin epidermal layer—the 0.03% AgNPs/PLA/Col1 dressing demonstrated notably thicker granulation tissue, well-aligned connective tissue fibers, and a dense capillary network indicative of advanced healing.

Masson’s trichrome staining further highlighted differences in collagen deposition and organization (Figure 8B). The 0.03% AgNPs/PLA/Col1 group exhibited markedly higher collagen deposition (71.45 ± 1.77%) and organized collagen fiber bundles, indicative of mature extracellular matrix formation [60]. In contrast, the untreated control group displayed fragmented collagen fibers (51.10 ± 1.53%), while other treated groups exhibited intermediate collagen organization. These findings underscore the critical role of precisely calibrated AgNP concentrations in enhancing extracellular matrix maturation and remodeling during diabetic wound healing.

CD31 immunohistochemical staining revealed enhanced neovascularization in the 0.03% AgNPs/PLA/Col1 dressing group, characterized by densely distributed CD31-positive vascular structures throughout the regenerated wound bed (Figure 8B). Enhanced angiogenesis directly correlates with superior granulation tissue maturity and collagen fiber alignment, as functional capillary networks supply essential oxygen and nutrients necessary for cellular proliferation and extracellular matrix remodeling [61]. In contrast, the untreated control group showed sparse CD31-positive expression, consistent with impaired angiogenesis typically observed in diabetic wound pathology.

### 3.7. Mechanistic Insights and Clinical Significance

The optimized 0.03% AgNPs/PLA/Col1 dressing demonstrated significant therapeutic efficacy through a multimodal mechanism, addressing several critical pathophysiological aspects inherent in diabetic wound healing. Specifically, green-synthesized AgNPs exhibited robust antibacterial activity, effectively reducing microbial colonization and potentially mitigating the chronic inflammatory response frequently observed in infected diabetic wounds. The electrospun nanofiber matrix provided a biomimetic architecture closely resembling native extracellular matrix, effectively supporting cellular infiltration, proliferation, and tissue regeneration. Furthermore, the incorporation of AgNPs noticeably improved collagen fiber deposition and alignment, directly addressing the impaired extracellular matrix remodeling and irregular collagen organization commonly associated with diabetic wounds.

Moreover, the controlled release of Ag^+^ ions from the nanofiber scaffold was correlated with enhanced angiogenesis, likely mediated via increased vascular endothelial growth factor expression. This mechanism significantly contributed to the formation of dense, functional capillary networks essential for supplying oxygen and nutrients within the hypoxic diabetic wound environment.

This integrated, multifunctional strategy offers distinct advantages over conventional single-target interventions, simultaneously addressing infection control, inflammation modulation, extracellular matrix regeneration, and angiogenesis. Notably, the wound closure rate of 94.62% observed in our diabetic rat model substantially exceeds previously reported outcomes for comparable interventions [62]. Critically, the identification of a precise therapeutic window (0.03% AgNPs) optimized for maximal antimicrobial efficacy without compromising cellular biocompatibility directly addresses a fundamental challenge in translating nanomedicine-based therapies into clinical practice. Consequently, our findings present a promising and translationally relevant platform, demonstrating significant clinical potential for the effective management of diabetic wounds.

## 4. Conclusions

This study successfully developed a green synthesis method for AgNPs utilizing *A. argyi* extract and subsequently incorporated these nanoparticles into bioactive nanofibrous dressings. Through systematic multi-parameter optimization, we produced monodisperse, spherical AgNPs with an average diameter of 6.76 ± 0.27 nm and a face-centered cubic (FCC) crystalline structure. HPLC-MS analysis verified that flavonoids and phenolic acids within the *A. argyi* extract served as the primary reducing and stabilizing agents during nanoparticle synthesis. The synthesized AgNPs demonstrated concentration-dependent antibacterial efficacy, achieving minimum inhibitory concentrations of 8 μg/mL against *E. coli* and 32 μg/mL against *S. aureus*. Importantly, these nanoparticles exhibited excellent biocompatibility with L929 fibroblast cells at concentrations ≤ 8 μg/mL. In the diabetic rat wound model, the optimized 0.03% AgNPs/PLA/Col1 dressing exhibited remarkable therapeutic properties mediated by a dual-functional mechanism. Specifically, the dressing provided sustained antimicrobial protection via controlled Ag^+^ release while simultaneously facilitating enhanced tissue regeneration through improved collagen deposition, extracellular matrix remodeling, and neovascularization.

Although the observed dual-functional mechanism presents highly promising results, further research is necessary to elucidate the precise molecular pathways underlying AgNP-induced tissue regeneration. Additionally, comprehensive evaluation of the long-term biological effects associated with sustained AgNP release and nanofiber dressing degradation in vivo must be undertaken to fully establish the clinical potential and safety of this innovative therapeutic platform.

## Figures and Tables

**Table 1:** Optimization conditions and UV-vis spectrum results in AgNP synthesis.

Sample No.	Parameter	Condition	Variable	UV (nm)
1	Ratio of *A.argyi* to AgNO_3_ (*v*/*v*)	AgNO_3_: 4 mM	1:1	415.5
		*A. argyi* extract: 4 mg/mL	1:2	414
		pH: 8	1:3	416
		Time: 40 min	1:4	416
		T: 50 °C	1:5	416.5
2	Concentration of AgNO_3_ (mM)	*A.argyi*:AgNO_3_: 1:1(*v*/*v*)	2	413
		*A. argyi* extract: 4 mg/mL	4	414
		pH: 8	6	416
		Time: 40 min	8	420.5
		T: 50 °C	10	422
3	Concentration of plant extract (mg/mL)	*A.argyi*:AgNO_3_: 1:1 (*v*/*v*)	1	418.5
		AgNO_3_: 6 mM	2	416.5
		pH: 8	4	415.5
		Time: 40 min	6	415.5
		T: 50 °C	8	415
4	pH	*A.argyi*:AgNO_3_: 1:1 (*v*/*v*)	5	321
		AgNO_3_: 6 mM	6	425.5
		*A. argyi* extract: 8 mg/mL	7	417
		Time: 40 min	8	413.5
		T: 50 °C	9	412.5
5	Reaction time (min)	*A. argyi*:AgNO_3_: 1:1 (*v*/*v*)	15	415
		AgNO_3_: 6 mM	30	414.5
		*A. argyi* extract: 8 mg/mL	45	415
		pH:8	60	414.5
		T: 50 °C	75	414
6	Temperature (°C)	*A.argyi*:AgNO_3_: 1:1 (*v*/*v*)	25	412.5
		AgNO_3_: 6 mM	40	414.5
		*A. argyi* extract: 8 mg/mL	60	414
		pH: 8	80	415.5
		Time: 60 min	90	411.5

## Data Availability

The original contributions presented in this study are included in the article; further inquiries can be directed to the corresponding authors.

## Abbreviations

The following abbreviations are used in this manuscript:

AgNPs	Silver nanoparticles
FCC	Face-centered cubic
PLA	Polylactic acid
Col1	Collagen type I
*A. argyi*	*Artemisia argyi*
UV-vis	Ultraviolet-visible
TEM	Transmission electron microscopy
SEM	Scanning electron microscopy
XRD	X-ray diffraction
EDS	Energy dispersive X-ray spectroscopy
FTIR	Fourier-transform infrared spectroscopy
PDI	polydispersity index
DLS	dynamic light scattering
*E. coli*	*Escherichia coli*
*S. aureus*	*Staphylococcus aureus*
HPLC-MS	High-performance liquid chromatography coupled with mass spectrometry
H&E	Hematoxylin and eosin
HCl	Hydrochloric acid
NaOH	Sodium hydroxide
DMEM	Dulbecco’s Modified Eagle Medium
FBS	Fetal bovine serum
CCK-8	Cell Counting Kit-8
LB	Luria–Bertani
OD	Optical density
OD_600_	Optical density at 600 nm
MIC	Minimum Inhibitory Concentration
MBC	Minimum Bactericidal Concentration
NIH	National Institutes of Health
SD	Sprague–Dawley
STZ	Streptozotocin
ANOVA	Analysis of variance.
